# Self-management of social well-being in a cross-sectional study among community-dwelling older adults: The added value of digital participation

**DOI:** 10.1186/s12877-021-02482-6

**Published:** 2021-10-11

**Authors:** Mariska MJ Scheffer, Juliane Menting, Hennie R Boeije

**Affiliations:** 1 Netherlands Institute for Health Services Research (Nivel), Utrecht, The Netherlands; 2 Department of Care and Participation of people with chronic conditions, Netherlands Institute for Health Services Research (Nivel), PO Box 1568, BN 3500 Utrecht, The Netherlands

**Keywords:** Chronic condition, Internet usage, Older people, Self-management abilities, Social needs

## Abstract

**Background:**

This study aimed to examine associations between self-management abilities and digital participation among community-dwelling older adults with chronic conditions in the Netherlands.

**Methods:**

The study utilized a cross-sectional design. Community-dwelling older adults were sampled from a Dutch nationwide panel study performed in October and November of 2019. We selected all adults of 65 years and above who had one or more chronic diseases (*n* = 1,656). Self-management was measured by six abilities (e.g., investing in resources for long-term benefits and taking care of a variety of resources), whereas digital participation was estimated with the frequency of four social internet uses (e.g., using social network websites and calling digitally).

**Results:**

When predicting self-management abilities from digital participation, hierarchical multiple regression analysis determined statistically significant and positive relationships, in particular for e-mailing (β = 0.21; *p* < .001) and meeting new people online (β = 0.07; *p* < .05). Correlation analyses showed that highest associations were found between internet usage and the self-management abilities ‘taking initiative’ (*r* = .23; *p* < .001) and ‘being self-efficacious’ (*r* = .21; *p* < .001). Moreover, the study confirmed that higher age (β = -0.13; *p* < .001) and increased severity of disability (β = -0.12; *p* < .01) negatively impact abilities for self-management.

**Conclusions:**

These findings suggest that internet usage and self-management abilities are positively related in the older adult population. Further research should be undertaken to examine the links between self-management abilities and digital participation more closely.

## Background

It is well established that social resources, such as family ties and presence of neighbours, have a profound impact on the development of social well-being in later life [1]. Moreover, older adults evaluate social connectedness as one of the most important determinants of successful aging [2]. Nevertheless, achieving social well-being becomes more challenging across one’s life span, as the availability of resources tends to decline with age [3]. This is especially the case for community-dwelling older adults suffering from one or more chronic conditions, who are likely to have fewer opportunities to maintain and develop new close relationships [4, 5].

There is a growing body of literature that recognizes the importance of older adults’ self-management abilities to manage social resources in such a way that well-being is maintained and restored when lost [6–8]. Self-management abilities, the behavioural and cognitive abilities to sustain well-being in later life, are functional in the fulfilment of basic social needs, namely status, behavioural confirmation and affection [8, 9]. Self-management for well-being can be distinguished in six abilities: (1) taking initiative, (2) investing in resources for long-term benefits, (3) taking care of resource multi-functionality, (4) being self-efficacious, (5) taking care of a variety of resources, and (6) having a positive frame of mind [8]. For example, when someone is taking the initiative to remain in good contact with significant others, this is expected to contribute to the fulfilment of affectional needs [8]. Hence, strengthening the core self-management abilities for well-being can aid in the prevention of age-related social problems among older adults.

Factors found to be influencing social well-being among older adults have been explored in various studies [3]. Previous research has established that more general resources, for instance lifestyle, educational level and living situation, can be beneficial for fulfilling social needs [3]. In addition, it has been observed that access to information and communication technologies (ICTs), such as computers, tablets or mobile phones, are associated with greater life satisfaction, lower depression and less feelings of loneliness [10]. Moreover, various communication domains exist, including chatrooms, newsgroups, message boards, e-mailing or instant messaging [11]. Accordingly, when ICTs are utilized for communication purposes, such as receiving and providing peer support, family related discussions and reaching out to new people, it has the potential to enhance social well-being [11, 12].

To date, the majority of older adults has gained access to internet-based communication services in the Netherlands [13]. Additionally, internet usage for social purposes within this group is expected to expand [14]. Although digital participation does not substitute all kinds of face-to-face activities, the internet may be a useful instrument to support self-management abilities when social resources are declining. Notwithstanding, little is known regarding the relationship of digital participation for social purposes and peoples’ abilities of self-management. Therefore, this cross-sectional research investigated associations between self-management abilities and social internet usage, such as visiting social networking sites and digital calling, among community-dwelling older adults.

## Methods

### Study population

The research focuses on older adults with at least one chronic condition in the Netherlands. The study made use of the ‘National Panel of the Chronically ill and Disabled’ (NPCD) [15]. This nation-wide panel collects data on the experiences and consequences of people with a chronic disease and/or physical disability from the perspective of the patient. Twice a year, panel members are asked to fill in a postal or online questionnaire including various health-related topics. Yearly, panel members are selected to replace members who either withdrew or had participated for the maximum term of four years. People with chronic illness are recruited from random samples of general practices that are drawn from the Dutch Database of General Practices. Eligibility criteria required individuals to have a diagnosis of a somatic chronic condition (e.g., diabetes, chronic obstructive pulmonary disease, cancer, cardiovascular disease, musculoskeletal disorders) according to population wide survey data. Exclusion criteria include: age ≤ 15 years, being institutionalized, unaware of diagnosis, life expectancy < 6 months according to the GP and insufficient mastery of the Dutch language. Chronic diseases were diagnosed by a certified medical doctor and were defined by the International Classification of Primary Care (ICPC). For the present study, data on the topics on self-management and internet usage were selected from the panel study performed in October and November of 2019. Participants of the panel study were included if they had at least one somatic chronic disease, and were 65 years or above. The NPCD Program Committee approved the present research.

### Measures

#### Self-management abilities

Self-management abilities were assessed with the short version of the Self-Management Ability Scale (SMAS-S), which is proven to be a reliable instrument [6, 16], and has been evaluated in a large sample of community-dwelling elderly people, 65 years of age and above [8]. This instrument consists of 18 items, and includes six subscales evaluating the ability to (1) take initiatives (e.g., “how often do you take the initiative to keep yourself busy?”), (2) invest in resources for long-term benefits (e.g., “do you devote some time and attention to those who are dear to you in order to maintain good contact?”), (3) self-efficaciously manage resources (e.g., “are you able to have friendly contacts with others?”), (4) ensure resource multi-functionality (e.g., “the activities I enjoy, I do together with others”), (5) maintain variety in resources (e.g., “how many hobbies or activities do you have on a regular basis?”) and (6) adopt a positive frame of mind (e.g., “when you have a bad day, how often do you think that things will be better tomorrow?”). Each subscale ranges from 1 to 6, with higher scores indicating higher levels of self-management abilities.

#### Digital participation

Digital participation was assessed by internet usage for social purposes. The indicators for social internet usage that were measured in the study comprised of four items: (1) visiting social networking sites; (2) meeting new people online; (3) maintaining contact via e-mail; and (4) digital calling. Answering categories for these questions were (1) rarely or never; (2) less than once a month; (3) at least once a month; and (4) at least once a week. Indicators for social internet usage were dichotomised into ‘minimal internet usage’ (rarely or never/less than once a month) and ‘frequent internet usage’ (at least once a month/week).

#### Background characteristics

The study controlled for confounding effects of background characteristics. These variables may be associated with levels of self-management abilities and social internet usage [17, 18]. The factors that were taken into consideration were age, gender, living situation (live alone, living with others), education (low, middle and high education level), number of somatic chronic conditions (one, two or more), and severity of disability (none to moderate disability, mild to severe disability).

### Statistical analysis

First, we assessed the reliability of the SMAS-S and demonstrated that the internal consistency of the overall scale in this study is high with a Cronbach’s alpha coefficient of 0.89. The internal consistency of the individual subscales ranged from 0.68 to 0.82. Second, descriptive statistics were reported for self-management abilities, social internet usage and background characteristics. Third, bivariate correlation analysis was conducted to test potential correlations between self-management abilities and digital participation, and with relevant background variables. Pearson correlation was applied for describing correlations between self-management ability scores. For correlations between internet uses and self-management abilities, a Spearman correlation was applied. Lastly, hierarchical linear regression was used with self-management abilities as a continuous outcome variable and internet uses as potential predictor variables. Background characteristics were taken into consideration to control for confounding effects. To treat missing data, complete case analysis was employed. Results were considered statistically significant at *p* < .05. Statistical analysis was performed using STATA (version 15.0, StataCorp, College station, Texas).

## Results

### Descriptive statistics population sample

Table 1 presents the descriptive statistics for the study population. A total of 1,656 older adults with chronic diseases participated in the study (76 % response rate). Approximately half of the sample (48.7 %) was male. The mean age was 73.8, with a standard deviation (SD) of 6.4 years. Most respondents had middle education (44.3 %), while respectively 29.2 % and 44.3 % had lower or higher education levels. The majority of respondents was living with others (71.2 %), had two or more somatic conditions (63.7 %) and reported none to moderate disability (59.2 %). Regarding digital participation, 43.3 % of the respondents visited social media platforms at least once a month. Moreover, 70.7 % made use of the internet for maintaining contact with others via email, 36.5 % for the purpose of digital calling, and 6.7 % employed the internet to meet new people. Among the six self-management abilities, the highest scores were found for the abilities investment behaviour (mean score 4.4; SD 0.9; range 1–6) and self-efficacy (mean score 4.2; SD 0.6; range 1–5). In contrast, the lowest score was for having a variety of resources (mean score 3.3; SD 0.9; range 1–6).

**Table 1:** Descriptive Statistics of Background Characteristics, Self-management Abilities and Digital Participation among Community-dwelling Older Adults

Characteristic	Mean (SD) or frequency	N
Gender		
Female	51.3 %	849
Male	48.7 %	807
Age	73.8 (6.4)	1,656
Education ^a^		
Low	29.2 %	461
Middle	44.3 %	700
High	26.5 %	419
Living situation		
Alone	28.8 %	466
With others	71.2 %	1,153
Number of somatic conditions		
One	36.3 %	489
Two or more	63.7 %	858
Severity of disability		
None to moderate	59.2 %	933
Mild to severe	40.8 %	642
Total score self-management	23.3 (3.9) (range 6–35)	1,379
Taking initiative	3.9 (0.9) (range 1–6)	1,552
Investment behaviour	4.4 (0.9) (range 1–6)	1,547
Variety of resources	3.3 (0.9) (range 1–6)	1,474
Multi-functionality	3.5 (1.0) (range 1–6)	1,525
Self-efficacy	4.2 (0.6) (range 1–5)	1,550
Positive frame of mind	3.9 (1.0) (range 1–6)	1,551
Internet user	85.0 %	1,347
Frequency of visiting social network sites		
Minimal usage	56.7 %	743
Frequent usage	43.3 %	568
Frequency of meeting new people		
Minimal usage	93.3 %	1,206
Frequent usage	6.7 %	86
Frequency of e-mailing		
Minimal usage	29.3 %	382
Frequent usage	70.7 %	923
Frequency of calling digitally		
Minimal usage	63.5 %	833
Frequent usage	36.5 %	479

^a^ High: high vocational education or university; Middle: intermediate or advanced general education or intermediate vocational training; Low: primary school or preparatory vocational training

### Associations between internet uses and self-management abilities

An overview of correlation analysis results is set out in Table 2. Findings indicate that overall self-management scores were significantly and positively associated with social internet usage (visiting social networking sites (*r* = .09; *p* < .01); meeting new people online (*r* = .14; *p* = < 0.001); maintaining contact via email (*r* = .28, *p* < .001); digital calling (*r* = .14; *p* < .001)). The strongest association was observed between maintaining contact via email and self-management. Each of the social internet usages had a significant and positive association with at least four out of six individual self-management abilities. High associations were found for the self-management abilities taking initiative and being self-efficacious. Internet usage for maintaining contact via email and digital calling were significantly and positively associated with all individual self-management abilities.

**Table 2:** Correlations between Self-management Abilities and Indicators for Social Internet Usage among Community-dwelling Older Adults

Characteristic	1	2	3	4	5	6	7	8	9	10
1. Overall self-management	-									
2. Taking initiative	0.77***	-								
3. Self-efficacy	0.73***	0.54***	-							
4. Investment behaviour	0.83***	0.60***	0.60***	-						
5. Variety of resources	0.73***	0.46***	0.47***	0.56***	-					
6. Multi-functionality	0.75***	0.51***	0.42***	0.55***	0.50***	-				
7. Positive frame	0.53***	0.27***	0.31***	0.30***	0.21***	0.18***	-			
8. Visiting social network sites	0.09**	0.08**	0.08**	0.09**	0.06*	0.04	0.08**	-		
9. Meeting new people	0.14***	0.12***	0.13***	0.10***	0.12***	0.11***	0.04	0.24***	-	
10. E-mailing	0.28***	0.23***	0.21***	0.20***	0.17***	0.20***	0.12***	0.21***	0.15***	-
11. Calling digitally	0.14***	0.16***	0.14***	0.10***	0.12***	0.09***	0.07**	0.17***	0.10***	0.23***

Note.*p < .05 **p < .01 ***p < .001

### Digital participation as predictor of self-management abilities

Table 3 presents the hierarchical multiple regression analysis that explains variation in older adults’ self-management abilities. The first step of the analysis, including all background variables, indicates that female older adults possess higher levels of self-management compared to men (β = 0.09; *p* < .01). Furthermore, higher age (β = -0.18; *p* < .001), more severe disabilities (β = -0.15; *p* < .001) and lower education (β = -0.11; *p* < .01) were significantly and negatively associated with self-management abilities. In the second step, the explained variance increased when adding internet variables to the model. In contrast to the remaining background variables, the relationship between level of education and self-management abilities was no longer significant. Moreover, two out of four indicators for digital participation were positively and significantly associated with levels of self-management abilities. Older adults who maintain contact via e-mail (β = 0.21; *p* < .001) and meet new people online (β = 0.07; *p* < .05), seem to be better self-managers of their social well-being.

**Table 3:** Hierarchical Multiple Regression Analysis predicting Self-management Abilities among Community-dwelling Older Adults

	Model 1				Model 2		
**Variable**	***B***	***SE B***	***β***		***B***	***SE B***	***β***
*Step 1: background variables*							
Gender (female)	2.09	0.72	0.09**		2.19	0.77	0.10**
Age (higher)	-0.34	0.06	-0.18***		-0.26	0.07	-0.13***
Educational level ^a^							
Low	-2.76	0.87	-0.11**		-1.03	0.98	-0.04
High	0.32	0.82	0.01		-0.52	0.84	-0.02
Living situation (with others)	-0.78	0.82	-0.03		-0.65	0.89	-0.03
Number of somatic conditions (two or more)	-0.55	0.73	-0.02		-0.58	0.79	0.03
Severity of disability (mild to severe)	-3.59	0.77	-0.15***		-2.79	0.83	-0.12**
*Step 2: internet variables*							
Visiting social network sites (frequent usage)					0.86	0.78	0.04
Meeting new people (frequent usage)					3.63	1.82	0.07*
E-mailing (frequent usage)					5.19	0.87	0.21***
Digital calling (frequent usage)					1.26	0.78	0.06
*N*	1,019				815		
R^2^	0.088				0.116		

*Note.* *** *p* < .001, ** *p* < .01, * *p* < .05

^a^ Middle education was used as a reference group

## Discussion

Drawing on a nationally representative sample of older adults with chronic conditions, this cross-sectional study set out to assess associations between digital participation (frequency of visiting social network sites, meeting new people, e-mailing and calling digitally) and self-management abilities for well-being. The results of this study show a strong association between internet usage and self-management abilities among older adults. The findings indicate that two internet uses are associated with levels of self-management abilities in particular. Frequently meeting new people online and maintaining contact via e-mail was significantly and positively associated with stronger self-management abilities. Maintaining contact via email may expand or replace traditional forms of social contact, when social relations are changing in later life. Moreover, in the face of declining social resources, meeting new people online may be especially important for self-management, preventing social isolation and loneliness among older adults [19]. Contrary to our expectations, participating in online calling and social media were not significantly associated with self-management abilities. This might be partly explained by the various effects of social media that have been identified by Pittman and Reich [20]. It has been shown that image-based platforms can positively affect levels of self-reported loneliness, while text-based platforms are unable to increase social well-being due to a lack of intimacy.

Regarding individual self-management abilities, strong associations were found between indicators for social internet usage and the abilities taking initiative and having self-efficacy. These results partly corroborate earlier observations, which presented a robust relationship between internet usage and older adults’ self-efficacy beliefs [21]. It is likely that digital participation enhances the opportunities to initiate meaningful contact with friends and family. Also, older adults may receive additional social support from others through the internet, which can lead to a stronger sense of efficacy. Social support is often seen as a potential mediator of social well-being, as it can establish emotional connections [11].

The study also provided additional insight into community-dwelling older adults’ levels of digital participation and self-management abilities for well-being. Among the six abilities for self-management, the highest scores were found for the abilities investment behaviour (mean score 4.4) and self-efficacy (mean score 4.2). These results reflect those of Nieboer et al. [22] in a sample of community-dwelling people aged 55 years and older (respectively 4.6 and 4.3). In contrast, older adults score the lowest for the ability of having a variety in resources, with a mean score of 3.3. This finding is also consistent with data obtained in other research in this field (mean score 3.6) [22]. Lower scores for variety of resources may be explained in view of the fact that older adults have smaller social networks than younger adults in general [23]. Nevertheless, this does not seem to have a negative impact on overall well-being, as the perception of relationship quality seems to be more relevant among older individuals, rather than the relationship quantity [23].

While controlling for confounding effects of background characteristics, significant associations between internet uses meeting new people online and maintaining contact via email, and self-management abilities were identified. Female gender, higher age and the severity of disability contributed importantly to the relationship between digital participation and self-management. The present study confirmed that older adults with higher age seem to have lower self-management abilities, in contrast to younger, older adults, which is in line with the self-management of abilities theory describing that self-management becomes more challenging in later life [6]. Interestingly, levels of self-management were higher for female older adults, compared to those of males. This may be related to the fact that female older individuals appear to have higher levels of participation in social group activities than male individuals [24]. Female individuals also seem to be better self-managers of their chronic condition [25, 26]. Additionally, older adults with more severe disabilities scored lower on the self-management scale, while the number of chronic conditions did not seem to have a significant impact. Considering that people living with both chronic disease and disability have an increased risk of poor health status and life dissatisfaction, compared to those living with chronic disease alone [27], may explain associations with poorer self-management [28].

This paper adds to the growing body of research on self-management abilities in older patients. The study employed a large panel to examine the relationship between digital participation and self-management abilities among community-dwelling older adults with chronic disease(s). Participants in the panel suffer from various chronic diseases, which make them representative of this population living in the Netherlands. The use of validated scales, including the SMAS-S and physical disability measures, also adds to the strength of this study. Furthermore, the high response rate of the survey (76 %) within this large panel allows for generalization of the study findings. Nevertheless, several issues should be addressed when interpreting the findings. Although the response rate was relatively high, we cannot exclude the possibility of nonresponse bias. Moreover, the study is limited to four indicators of internet usage (frequency of visiting social network sites, meeting new people, e-mailing and calling digitally) to examine the associations between self-management and digital participation, while other indicators for digital participation may be more or less relevant. Also, the cross-sectional nature of the study cannot exclude a reverse causation. It might be the case that self-management abilities also positively affect older adults’ internet use. For example, it may be that better self-managers are more confident and take more initiatives to participate digitally. Earlier studies have established relationships between self-efficacy and technology use. Findings showed that older adults with high levels of self-efficacy were more likely to make use of the internet for social needs than those who had a lower self-efficacy [20, 29]. Accordingly, it may be that other abilities for self-management influence older adults’ perceptions of internet use as well. Lastly, other factors may also moderate the relationship between internet usage and self-management abilities, for instance health status and the level of health literacy of older adults [28, 30]. In spite of its limitations, the study certainly adds to our understanding of the wide range of factors associated with self-management abilities for social well-being. Future research should investigate the impact of digital participation with a broader concept of internet usage, including participation in online activities and information seeking. Moreover, additional studies using qualitative methods could provide more in-depth information, and shed light on the direction of the established relationship between the internet and self-management abilities.

Taken together, this study shows that digital participation, in particular maintaining contact via e-mail and meeting new people, is positively associated with self-management abilities among community-dwelling older adults. Further research is needed to examine the links between self-management abilities and digital participation more closely. Male older adults with older age and physical disability may require additional attention when supporting self-management abilities.

## Data Availability

The dataset used during the current study is available from the corresponding author on reasonable request.

## Abbreviations

ICTs: Information and Communication Technologies
NPCD: National Panel of the Chronically ill and Disabled
SMAS-S: Self-Management Ability Scale Short version
